# Evidence of recent evolutionary selection at eQTLs for APOE

**DOI:** 10.1002/alz.092921

**Published:** 2025-01-03

**Authors:** Timothy H Ciesielski, Giuseppe Tosto, Razaq O Durodoye, Farid Rajabli, Rufus O. Akinyemi, Margaret A Pericak‐Vance, Jonathan L. Haines, Alzheimer’s Disease Genetics Consortium (ADGC), Scott M Williams

**Affiliations:** ^1^ Case Western Reserve University, Cleveland, OH USA; ^2^ Taub Institute for Research on Alzheimer’s Disease and the Aging Brain, Vagelos College of Physicians and Surgeons, Columbia University, New York, NY USA; ^3^ John P. Hussman Institute for Human Genomics, University of Miami Miller School of Medicine, Miami, FL USA; ^4^ Neuroscience and Aging Research Unit, Institute of Advanced Medical Research and Training, College of Medicine, University of Ibadan, Ibadan Nigeria; ^5^ 1501 NW 10th Avenue, Miami, FL USA; ^6^ Department of Population and Quantitative Health Sciences, Institute for Computational Biology, Case Western Reserve University, Cleveland, OH USA; ^7^ –, –, PA USA

## Abstract

**Background:**

Late‐onset Alzheimer Disease (LOAD) risk and prevalence differ by ancestral group. Furthermore, the frequency of *APOE‐4* and its effect size on LOAD risk also differ by ancestry group. If these patterns are a function of evolutionary history, we may find ancestry group‐specific evidence of recent selection at the *APOE* locus or *APOE* eQTLs. This could provide us with additional insights into APOE function, specifically its pleiotropic effects and ethnic specific mechanisms of disease.

**Methods:**

We used chromosome 19 sequence data from 2,504 unrelated individuals in 26 populations from the latest release of the 1000 Genomes Data (phased 30x coverage build 38 data), and we ran separate selection analyses in each population. We calculated two selection measures based on Extension of Haplotype Homozygosity (EHH): iHS (integrated haplotype score) and nSL (number of segregating sites by length). Scores were then normalized in bins of similar allele frequency (AF) to account for frequency effects on EHH. Loci in the top 1% of |iHS| and |nSL| scores were considered evidence of selection.

**Results:**

Among the two SNPs defining the *APOE isoforms* and 27 SNPs defining *APOE* EQTLs (29 sites in total) we observed 9 *APOE* eQTLs in the top 1% of iHS and three in the top 1% of nSL. Two SNPS demonstrated evidence of selection in both scores: rs1628394 in Kinh Vietnamese (in a SIPA1L3 intron) and rs2909088 in Han Chinese (in a ZFP30 intron). Neither of the *APOE* SNPs showed evidence of selection in any population.

**Conclusions:**

We identified evidence of recent natural selection in *APOE* eQTLs in two East Asian populations: rs1628394 in Kinh Vietnamese and rs2909088 in Han Chinese. Prior work has established that rs1628394 is nominally associated with LOAD (p = 2.45 × 10^‐3^) and the genome Aggregation Database indicates that it is common in Africans (AF = 0.63), less common in Europeans (AF = 0.33), and least common in East Asians (AF = 0.13). The second SNP, rs2909088, is not associated with LOAD, but it is also less common in East Asians (AF_European_ = 0.87, AF_African_ = 0.69, and AF_EastAsian =_ 0.38). Recent selection at rs1628394 may be enhancing the impact of *APOE4* on LOAD development in Kinh Vietnamese.

